# Resonant vibrations of a non-ideal gyroscopic rotary system with nonlinear damping and nonlinear stiffness of the elastic support

**DOI:** 10.1016/j.mex.2022.101993

**Published:** 2023-01-04

**Authors:** Zharilkassin Iskakov, Nutpulla Jamalov

**Affiliations:** a Joldasbekov Institute of Mechanics and Engineering, Almaty, Kazakhstan; b Al Farabi Kazakh National University, Joldasbekov Institute of Mechanics and Engineering, Almaty, Kazakhstan

**Keywords:** Gyroscopic rotor, Nonlinear damping, Non-ideal source, A method for numerical solution of differential equations of motion of nonideal rotary system, when energy source performance characteristic is unknown

## Abstract

When motor performance characteristic is unknown, non-linear differential equations of motion of nonideal gyroscopic rigid rotary system with nonlinear cubic damping and nonlinear stiffness of the elastic support turn out to be numerically unsolvable.

• In this case, the method uses the motor performance characteristic expression found from the frequency equation of forced stationary oscillations based on assumption that the angular acceleration is many times less than the square of the angular speed of rotation, replacing the stationary rotation angular speed with the shaft rotation angle derivative.
• The method correctness is evidenced by a good consistency between the rotor motion equation numerical solution results and the analytical solution results, and by the nonlinear cubic damping of the shaft angular coordinate oscillograms obtained by direct simulation, as well as by comparison with the results of numerical simulation for a straight-line DC motor performance characteristic.
• The method limitations are that it is used for the first approximation and weak nonlinear oscillations in the resonance region, where the shaft rotation speed is of the order of the oscillating system natural frequency.

## Graphical abstract

**Figure:**
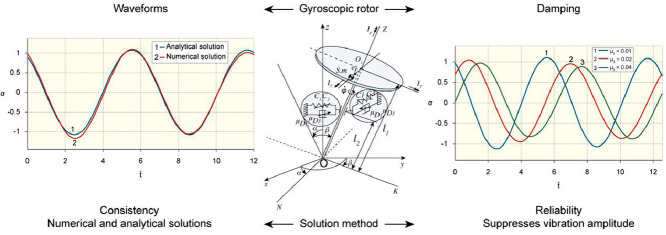

Specifications Table

**Table:** 

Subject area:	Engineering
More specific subject area:	Mechanical Engineering, Rotordynamisc, Nonlinear Damping
Method name:	A method for numerical solution of differential equations of motion of nonideal rotary system, when energy source performance characteristic is unknown
Name and reference of original method:	N.A.
Resource availability:	https://data.mendeley.com/datasets/4fwzjpxy2w

## Introduction

Despite the sufficient amount of literature devoted to the study of the dynamics of rotary machines, there are many problems associated with the vibrations arising during the acceleration and running down of the machine, and its stationary mode of operation. Their sources are the uneven distribution of mass, malfunctions in the support structure, wear of the disk, support bearings and shaft and their fatigue, the actions of other potential external forces. Loads on the machine, which create oscillatory processes when passing through critical speeds and natural frequencies, can lead to serious failures, affect performance characteristics and even destroy the structure of the machine. Consequently, stabilization of the motion of the rotary machine during its start-up, shutdown of the engine and operation, by damping unwanted vibrations, becomes relevant.

Most recently the number of research of vibration isolators has scaled up massively. The reason is that the vibration isolation materials of the machine are nonlinear by nature [1], [2], [3], and along with it, it is the nonlinear vibration isolation model that shows a wider stable speed range and therefore it is highly efficient than linear damping.

Experimental studies in [1], [2], [3] confirmed that the restoring and damping properties of viscoelastic materials should be described in nonlinear models and it was found that rubber insulators have both nonlinear stiffness and nonlinear damping. Subsequently, nonlinear shock absorbers began to be studied both by analytical and numerical methods, as well as by experimental methods [4], [5], [6], [7], [8], [9], [10], [11], [12], [13], [14], [15], [16], [17], [18], [19].

In [4], a comprehensive study was carried out on the effect of linear damping and nonlinear cubic damping on the force and displacement of the vibration isolators (transmissibilities). Comparative analysis of the transmission curves showed the advantages of nonlinear viscous damping. It is known that the nonlinear stiffness of the insulator material contributes to the transfer of force and the movement of changeability beyond the resonance region. Linear damping and nonlinear damping significantly suppress the resonant amplitude of oscillations, eliminate the jumping effect and harmonics caused by the nonlinear stiffness component, and only nonlinear cubic damping can suppress the vibration amplitudes in the above resonant frequency domains [5].

Along with the development of modeling of viscous linear damping and viscous cubic nonlinear damping of a vibrating system model with one degree of freedom (SDOF), the use of viscoelastic rubber materials in the rotor dynamics has also increased altogether.

In [6,7], in rotary bearing systems, the use of viscoelastic support was useful for attenuating noise and vibration. In [8], an example of the use of viscoelastic support in rotary bearing systems is flexible rubber supports. In the article [9], its dynamic characteristics and the characteristics of the transmitted force are investigated by direct simulation of a flexible rotor shaft-disk system supported by a suspension system with nonlinear stiffness and damping. Theoretical results obtained here are confirmed by experimental studies carried out in the article [10]. In it, the vibration of the rotor with a flexible support containing springs or rubber sheets with rotor vibration with a rigid support base is compared in the experimental single-disk rotor system supported by ball bearings at both ends. The works [11], [12], [13], [14], [15] are devoted to the study of the combined effect of linear and nonlinear cubic damping on resonance and behind it stationary and non-stationary oscillations, and on the stability of the movement of a gyroscopic rigid rotor with linear rigidity and nonlinear cubic rigidity of an elastic support. In the case of a rigid nonlinear characteristic of an elastic support, it is proved that if linear viscous damping merely shifts the lower boundary of the instability region towards high rotation speeds, then nonlinear cubic damping narrows this region on all sides until it is completely eliminated [12], [13], [14].

In [12,13], it was experimentally confirmed that joint linear and nonlinear cubic damping of an elastic support made of rubber material can significantly reduce the vibration level of the rotor not only in the resonance region of the main harmonic and beyond it, but also eliminates vibrations of significant magnitude with jumping effects in the range of high shaft rotation speeds. It is known that quadratic nonlinearity of damping has a weak effect on the amplitude of the response of the gyroscopic rotor to suppress its vibration and on its stability [18,19]. Then, taking into account [1], [2], [3], it remains to guess that the suppression of significant elevations of the experimental amplitude frequency response is the result of the effect of the cubic nonlinear component of damping with the existing linear damping.

Another advantage of nonlinear cubic damping is that it not only expands the area of vibration isolation, but is also used to control the resonant oscillations of the rotor with large amplitudes and to exit the area of instability with a jumping effect or to attenuate and eliminate this effect [15].

One of the known models of nonlinear damping is phenomenological, i.e. proportional to the n-th degree of velocity, adopted in [4], [5], [6], [7], [8], [9], [10], [11], [12], [13], [14], [15], [16], [17], [18], [19] and many other works, for vibrating systems with small nonlinearities, close to linear systems. The results of theoretical studies of the responses of these systems are satisfactorily consistent with the results of experimental work [9], [10], [11], [12], [13]. There is also a more widely used and experimentally verified model, where the nonlinear damping is proportional to the product of the displacement square multiplied by the velocity, and the basic nonlinear nature of the damping is determined depending on the amplitude of oscillations [20], [21], [22].

There is a considerable amount of literature on the dynamics of rotor systems driven by non-ideal energy sources.

The power supply in a non-ideal source is limited, unlike in an ideal system, due to which the temporal variations of the driving torque and rotational speed are not known in advance, because they are influenced by the time variations of the dynamic load. In light of this fact, for the non-ideal systems, the governing equations corresponding to the ideal system must be completed with an additional equation describing the behavior (characteristics) of the energy source. In [23], the dynamic properties of various energy sources are described as functions of velocity torque.

The dynamic rotor system requires sufficient power / torque to accelerate under resonant conditions or critical speeds to operate at supercritical speed. Most practical drive sources can only provide limited power to the rotor system. Thus, if the power is insufficient to overcome the resonance, the rotor speed may not change in this resonance with an increasing amplitude of oscillations or it will take a long time to exit the resonance; and thereby damage the system [24], [25], [26]. However, if the motor is provided with sufficient excess power, then the rotor is accelerated to the such a speed while decreasing the vibration amplitude. This phenomenon of nonlinear jumping is called the typical Sommerfeld effect [27], [28], [29], [30], [31]. The jumping effect usually occurs near a critical speed. Usually, there is no certain value of the speed at which a nonlinear jump occurs, since when the movement accelerates, the jump occurs at a speed above the critical speed, when the movement slows down – below the critical speed. Resonant capture and missing operating speeds are typical features associated with the Sommerfeld effect [[24], [25], [26],[32], [33], [34], [35], [36], [37], [38]].

In [39] dynamics of the non-ideal oscillatory system in which the excitation is influenced by the response of oscillator is considered. Various types of non-ideal systems are investigated: linear and nonlinear oscillators with one or more degrees of freedom interacted with one or more energy sources.

The oscillations that appear in a non-ideal system during the passage through the critical rotational velocity can be analyzed using various methods. Various approximate analytical and numerical methods have been developed. We present some of them, which apply to nonideal nonlinear rotor systems with manifestations of the Sommerfeld effects without considering the chaos effect and related other nonlinear effects.

In [40], a dynamic model of a nonlinear cubic vibration isolation system is obtained, and the harmonic balance method (HBM) is used to solve the governing dynamic equation. In addition, a fourth-order Runge-Kutta numerical simulation is used to obtain the admissibility of the displacement of the system under study. To estimate the stiffness and damping parameters of a system with strong nonlinearities, a method [41] based on measurements of both jump frequencies and jump amplitudes of the system subjected to sinusoidal excitations is used. In many nonideal nonlinear oscillating systems, the relationship between amplitude and frequency is determined using the averaging method [13,15,42,43]. Numerical simulations are also presented to illustrate the results. A DC motor with a straight-line characteristic is used as a non-ideal power source. In [43], the behavior of the non-ideal rotor system is investigated near two simultaneously occurring resonances. The stability of the resonance response is also analyzed. It is known that different kinds of Sommerfeld effects are observed in nonideal rotor systems. In [44], the dynamics of an overhanging rotor system near the Sommerfeld effect modes are studied using a discrete and continuous shaft-rotor model in combination with a non-ideal motor drive model. The models are developed using a multi-energy domain modeling approach in the form of a bond graph model. This method is also used to study the transient dynamics of nonlinear rotating rotor systems driven by a nonideal DC motor [45], including a rigid rotor shaft with an unbalanced disk that is supported by flexible anisotropic supports/bearings [46].

The transient dynamics of a flexible rotating shaft with eccentricity driven by a nonideal DC motor with external and internal damping, the Sommerfeld effect characterized by a jump phenomenon, is also studied using the steady state amplitude obtained by the instantaneous power balance method and further verified by numerical simulation [47].

The above methods of dynamic analysis and analytical-numerical solution of differential equations of motion of non-ideal mechanical systems [40], [41], [42], [43], [44], [45], [46], [47] and given in other works are different, use non-ideal energy sources, mainly DC motors with certain characteristics. In contrast, the method of numerical solution of differential equations of nonideal fast-moving gyroscopic rigid rotor system [13] with nonlinear cubic damping and nonlinear rigidity of elastic support in the case of unknown motor performance characteristics in an extended version than in [13] is considered in this paper.

## Equation of motion

**Fig. 1:**
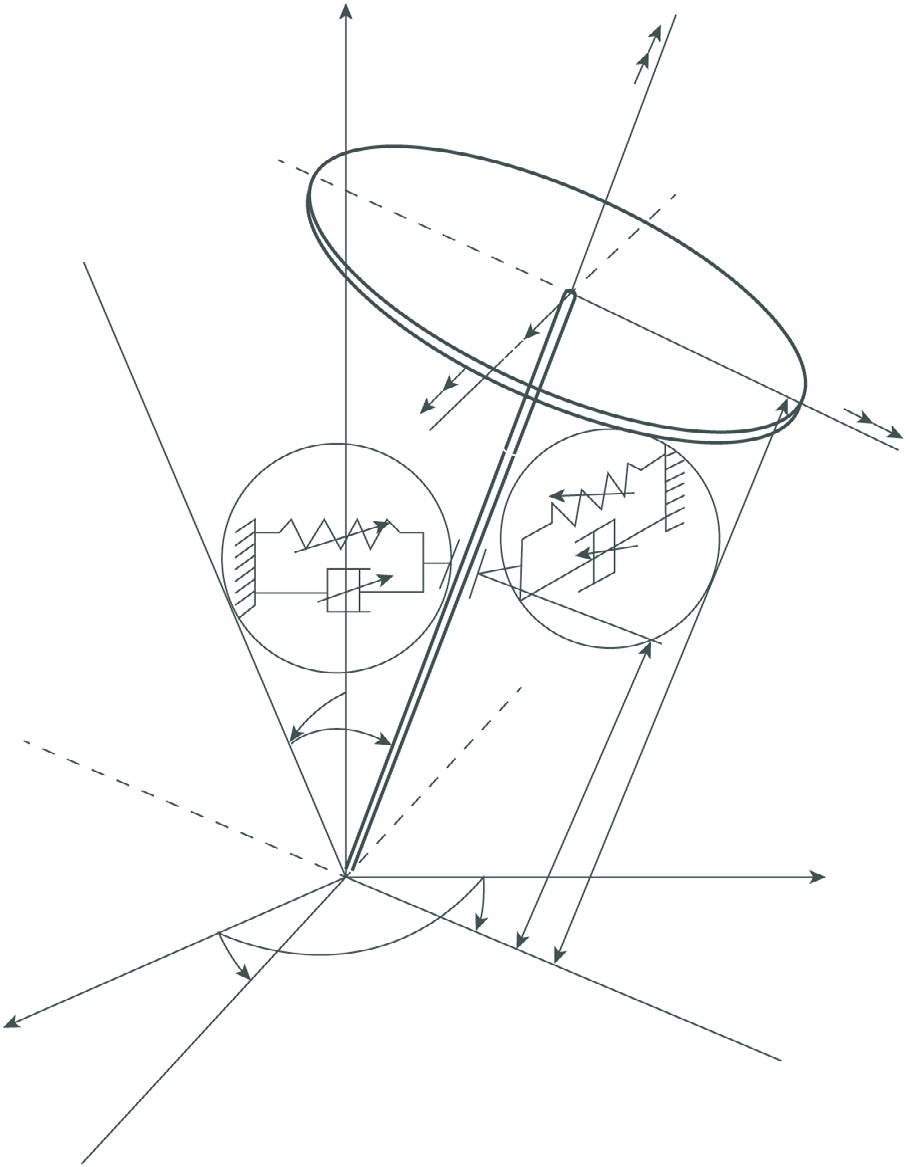
Structural diagram of the rotor.

The structural diagram of the gyroscopic rotor is shown in Fig. 1. The rotor receives excitation from a non-ideal energy source – a drive motor with a characteristic represented in a general form. The rotor disk has a mass *m*, a polar moment of inertia *I*_*p*_ and a transverse moment of inertia *I*_*T*_ and is fixed on the upper end of the shaft. The shaft with *l*_*1*_the length is installed vertically by means of lower universal hinged and upper elastic support, distance between supports *l*_*2*_. The rotational speed of the shaft about the axis of symmetry $\dot{\varphi }$ is such that the vertical rotor can be considered as a gyroscope. The upper support has restoring properties: linear stiffness $c_{1}$, nonlinear cubic stiffness $c_{3}$ and damping properties: linear damping $\mu_{D1}$, nonlinear cubic damping $\mu_{D3}$. Two coordinate systems are adopted: a fixed coordinate system Oxyz and a coordinate system rotating with the rotor ONKZ, the origin of which is the lower point of the shaft *O.* In the coordinate system, the position of the geometric center of the disk $O_{1}$ is determined by the coordinates x,y,z, and the position of the shaft and the rotor as a whole is determined by the Euler angles α, β and the angle of rotationφ φ=ωt. The angles α β are small and therefore, sinα≈α,sinβ≈β,cosα≈1,cosβ≈1 and the movement of the geometric center of the disk in the direction z can be neglected. In this case, the center of mass of the disk has the coordinates $x_{S}$ and $y_{S}$. We also assume that the linear eccentricity $e_{r}$ has the direction of the axis N of the coordinate system ONKZ.

Let us express the projections of the angular velocity of the rotor in the coordinate axes of the system ONKZ*,* the coordinates of the disk center of mass and the coordinates of the upper support through the angular coordinates α,β and φ, and find expressions for the kinetic energy, potential energy of the rotor, Rayleigh function and the projections of moments of forces acting on the system and the rotating motor moment. Substituting them into the Lagrangian equations of the second kind and using the following dimensionless parameters:

(1) $$\begin{array}{ccc} e & = & e_{r}/\left[l_{1}\left(1+{\bar{I}}_{T}\right)\right];\;l=l_{2}/l_{1};\bar{t}=t\omega_{0};{\bar{I}}_{p1}=I_{p}/\left[ml_{1}^{2}\left(1+{\bar{I}}_{T}\right)\right];{\bar{I}}_{T}=I_{T}/(ml_{1}^{2});\\ C_{1} & = & c_{1}/\left[m\omega_{0}^{2}\left(1+{\bar{I}}_{T}\right)\right];C_{3}=c_{3}l_{2}^{4}/\left[ml_{1}^{2}\omega_{0}^{2}\left(1+{\bar{I}}_{T}\right)\right];G=G/\left[ml_{1}\omega_{0}^{2}\left(1+{\bar{I}}_{T}\right)\right];\\ \mu_{1} & = & \mu_{D1}/\left[ml_{1}^{2}\omega_{0}\left(1+{\bar{I}}_{T}\right)\right];\mu_{3}=\mu_{D3}\omega_{0}/\left[ml_{1}^{2}\left(1+{\bar{I}}_{T}\right)\right];M=M_{m}/\left[ml_{1}^{2}\omega_{0}^{2}\left(1+{\bar{I}}_{T}\right)\right], \end{array}$$

where

(2) $$\omega_{0}=\sqrt{\frac{c_{1}l_{2}^{2}-Gl_{1}}{ml_{1}^{2}-\left(I_{P}-I_{T}\right)}}$$

- the natural frequency of the rotor system [13], where we obtain below equations of rotor motion:

(3) $$\begin{array}{ccc} & & \alpha^{\prime \prime }+\omega_{n}^{2}\alpha =e{\varphi^{\prime }}^{2}\cos\varphi -I_{P1}\varphi^{\prime }\beta^{\prime }-\mu_{1}\alpha^{\prime }-\mu_{3}{\alpha^{\prime }}^{3}-C_{3}\alpha^{3},\\ & & \beta^{\prime \prime }+\omega_{n}^{2}\beta =e{\varphi^{\prime }}^{2}\sin\varphi +I_{P1}\varphi^{\prime }\alpha^{\prime }-\mu_{1}\beta^{\prime }-\mu_{3}{\beta^{\prime }}^{3}-C_{3}\beta^{3},\\ & & \varphi^{\prime \prime }=\left[e\left(\alpha^{\prime \prime }\sin\varphi -\beta^{\prime \prime }\cos\varphi \right)-I_{P1}\left(\alpha^{\prime \prime }\beta +\alpha^{\prime }\beta^{\prime }\right)+M\left(\varphi^{\prime }\right)\right]/I_{P1}, \end{array}$$

where

(4) $$\sqrt{\frac{C_{1}l^{2}-G}{1+{\bar{I}}_{T}}}=\omega_{n}$$

- natural frequency of the rotor system vibration (3) where ${\bar{I}}_{T}\gg {\bar{I}}_{p}$.

In the system of Eq. (3) the dimensionless dynamic momentum of the energy source (motor) according to [12,30] is:

(5) $$M\left(\varphi^{\prime }\right)=L\left(\varphi^{\prime }\right)-q\varphi^{\prime }.$$

Where $L(\varphi^{\prime })$ - motor torque (characteristic), q - motor rotor rotation resistance coefficient.

When deriving the equations of motion (3), the following approximations were adopted. In the resonance and nearby region $\varphi^{\prime \prime }\ll {\varphi^{\prime }}^{2}$, and therefore, perturbations containing $\varphi^{\prime \prime }$perturbations having a parameter ${\bar{I}}_{p}\ll {\bar{I}}_{T}$ and perturbations having values of the second and higher orders of insignificance relative to α,β, their derivatives, and their combinations are small in comparison with perturbations whose amplitudes are proportional to the square of the angular velocity of the shaft. Therefore, they are discarded from Eq. (3).

The system (3) is a non-linear ordinary second-order differential equation relative to the angular coordinates α,β and φ.

## Solving equations of motion by averaging method

Consider a rotor system close to linear one. To apply the Bogolyubov-Krylov small perturbation theory method [13,15,33] to the solution of Eq. (3), the following limitations are adopted. The components of the moments of linear and cubic nonlinear damping force $\mu_{1}\alpha^{\prime },\mu_{1}\beta^{\prime }$ and $\mu_{3}{\alpha^{\prime }}^{3},\mu_{3}{\beta^{\prime }}^{3}$, as well as the moment of the cubic component of the elastic force $C_{3}\alpha^{3},C_{3}\beta^{3}$, the moment of inertia force of the mass imbalance $e{\varphi^{\prime }}^{2}\cos\varphi ,e{\varphi^{\prime }}^{2}\sin\varphi$ are considered small in comparison with the components of the moments of inertia force of vibration and the linear elastic force acting in the system. Projections of the moment of passive gyroscopic force $I_{P1}\varphi^{\prime }\alpha^{\prime }$ and $I_{P1}\varphi^{\prime }\beta^{\prime }$ can be considered small under the assumption that ${\bar{I}}_{p}\ll {\bar{I}}_{T}$. Let us also limit ourselves to the consideration of a fast-moving rotor: ${\varphi^{\prime }}^{2}\gg G$ and to the consideration of motion in an area where the frequency of forced oscillations Ψ is close to the frequency of free oscillations $\omega_{n}$. Therefore, we will look for solutions (3) in the form of replacement of variables [13]:

(6) $$\begin{array}{ccc} & & \alpha =A\cos\left(\varphi +\chi \right),\frac{d\alpha }{d\bar{t}}=-A\Omega \sin\left(\varphi +\chi \right),\\ & & \beta =A\sin\left(\varphi +\chi \right),\frac{d\beta }{d\bar{t}}=A\Omega \cos\left(\varphi +\chi \right),\Psi =\frac{d\varphi }{d\bar{t}}. \end{array}$$

Here, A,χ и Ψ are slowly changing functions of time$\bar{t}$, the main parameters of the oscillatory process:: A - oscillation amplitude, χ - angle of phase displacement between angular coordinates α or β and the moment of the perturbation force, Ψ- the frequency of the moment of the disturbance force or the angular velocity of rotation of the motor shaft.

Using the Krylov-Bogolyubov method [13,15,33], we obtain a system of equations relative to A,χ,Ψ, approximated solution of which is shown below

(7) $$\Psi =\Omega +\varepsilon U_{11}\left(\bar{t},\Omega ,a,\xi \right),A=a+\varepsilon U_{12}\left(\bar{t},\Omega ,a,\xi \right),\chi =\xi +\varepsilon U_{13}\left(\bar{t},\Omega ,a,\xi \right)$$

where $\varepsilon U_{11}\left(\bar{t},\Omega ,a,\xi \right),\varepsilon U_{12}\left(\bar{t},\Omega ,a,\xi \right)$ и $\varepsilon U_{13}\left(\bar{t},\Omega ,a,\xi \right)$ - small periodic functions of time $\bar{t}$, ε≪1– small parameter. The values of Ω,a,ξ are determined from the equations of the first approximation.

After averaging the equations of the first approximation equivalent to system (3), and equating their right-hand sides to zero, we arrive at the equations [13] to determine the frequency Ω

(8) $$L\left(\Omega \right)-q\Omega -\frac{\left(\mu_{1}+0.75\mu_{3}\omega_{n}^{2}a^{2}\right)}{\Omega }\omega_{n}^{2}a^{2}=0,$$

for determination of oscillation amplitude a:

(9) $$\left\{{\left(\mu_{1}\omega_{n}+0.75\mu_{3}\omega_{n}^{3}a^{2}\right)}^{2}+{\left[0.75C_{3}a^{2}+2\omega_{n}\left(\omega_{n}-\Omega \right)\right]}^{2}\right\}a^{2}={\left(e\Omega^{2}\right)}^{2}.$$

And expression for determination of oscillation phase *ξ* [13]

(10) $$\tan\xi =-\frac{\mu_{1}\omega_{n}+0.75\mu_{3}\omega_{n}^{3}a^{2}}{0.75C_{3}a^{2}+2\omega_{n}\left(\omega_{n}-\Omega \right)}.$$

Therefore, the refined angular coordinates α,β and φof the rotor can be written as [13]:

(11) $$\begin{array}{ccc} & & \alpha =a\cos\left(\Omega \bar{t}+\xi \right)+\varepsilon a_{11}\cos\left(\Omega \bar{t}+\xi_{11}\right)+\varepsilon a_{12}\cos\left(3\Omega \bar{t}+\xi_{12}\right)+\varepsilon a_{13}\cos\left(5\Omega \bar{t}+\xi_{13}\right),\\ & & \beta =a\sin\left(\Omega \bar{t}+\xi \right)+\varepsilon a_{21}\sin\left(\Omega \bar{t}+\xi_{12}\right)+\varepsilon a_{22}\sin\left(3\Omega \bar{t}+\xi_{22}\right)+\varepsilon a_{23}\sin\left(5\Omega \bar{t}+\xi_{23}\right),\\ & & \varphi =\Omega \bar{t}-\frac{1}{8\Omega }a^{2}\omega_{n}\left(\omega_{n}+\Omega \right)\sin2\left(\varphi +\xi \right), \end{array}$$

where constants $a_{11},a_{12},a_{13},a_{21},a_{22},a_{23},\xi_{11}\xi_{12},\xi_{13},\xi_{21},\xi_{22},\xi_{23}$ are determined from the following equations [13]

(12) $$\begin{array}{ccc} \varepsilon a_{11}\cos\left(\Omega \bar{t}+\xi_{11}\right) & = & \frac{e\left(\Omega^{2}+\bar{G}\right)}{8\omega_{n}\Omega }\cos\Omega \bar{t}-\frac{C_{3}a^{3}}{16\omega_{n}\Omega }\cos\left(\Omega \bar{t}+\xi \right)+\frac{\left(\mu_{1}+\mu_{3}\omega_{n}^{2}a^{2}\right)a}{8\Omega }\sin\left(\Omega \bar{t}+\xi \right),\\ \varepsilon a_{12}\cos\left(3\Omega \bar{t}+\xi_{12}\right) & = & \frac{e\left(\Omega^{2}+\bar{G}\right)}{8\omega_{n}\Omega }\cos\left(3\Omega \bar{t}+2\xi \right)+\frac{\left(\mu_{1}+\frac{7}{8}\mu_{3}\omega_{n}^{2}a^{2}\right)a}{8\Omega }\sin3\left(\Omega \bar{t}+\xi \right)\\ & & -\frac{5C_{3}a^{3}}{64\omega_{n}\Omega }\cos3\left(\Omega \bar{t}+\xi \right),\\ \varepsilon a_{13}\cos\left(5\Omega \bar{t}+\xi_{13}\right) & = & -\frac{\mu_{3}\omega_{n}^{2}a^{3}}{64\Omega }\sin5\left(\Omega \bar{t}+\xi \right)-\frac{C_{3}a^{3}}{64\omega_{n}\Omega }\cos5\left(\Omega \bar{t}+\xi \right),\\ \varepsilon a_{21}\sin\left(\Omega \bar{t}+\xi_{21}\right) & = & -\frac{e\left(\Omega^{2}+\bar{G}\right)}{8\omega_{n}\Omega }\sin\Omega \bar{t}+\frac{C_{3}a^{3}}{16\omega_{n}\Omega }\sin\left(\Omega \bar{t}+\xi \right)+\frac{\left(\mu_{1}+\mu_{3}\omega_{n}^{2}a^{2}\right)a}{8\Omega }\cos\left(\Omega \bar{t}+\xi \right),\\ \varepsilon a_{22}\sin\left(3\Omega \bar{t}+\xi_{22}\right) & = & \frac{e\left(\Omega^{2}+\bar{G}\right)}{8\omega_{n}\Omega }\sin\left(3\Omega \bar{t}+2\xi \right)-\frac{\left(\mu_{1}+\frac{9}{8}\mu_{3}\omega_{n}^{2}a^{2}\right)a}{8\Omega }\cos3\left(\Omega \bar{t}+\xi \right)\\ & & -\frac{3C_{3}a^{3}}{64\omega_{n}\Omega }\sin3\left(\Omega \bar{t}+\xi \right),\\ \varepsilon a_{23}\sin\left(5\Omega \bar{t}+\xi_{23}\right) & = & \frac{\mu_{3}\omega_{n}^{2}a^{3}}{64\Omega }\cos5\left(\Omega \bar{t}+\xi \right)-\frac{C_{3}a^{3}}{64\omega_{n}\Omega }\sin5\left(\Omega \bar{t}+\xi \right). \end{array}$$

Then rotor shaft oscillation frequency [13] is

(13) $$\Psi =\frac{d\varphi }{d\bar{t}}=\Omega -\frac{1}{4\Omega }a^{2}\omega_{n}\left(\omega_{n}+\Omega \right)\cos2\left(\Omega \bar{t}+\xi \right).$$

## Method details

### Solving equations of motion by direct simulation

For direct simulation of the rotor dynamics by Eq. (3) and comparison of its results with the results of analytical studies, when energy source characteristic is unknown, we use the method, the essence of which is described below.

For weak nonlinear oscillation, assuming that $\varphi^{\prime \prime }\ll {\varphi^{\prime }}^{2}$, the angular speed of stationary shaft rotation Ω can be replaced with $\varphi^{\prime }$. Then, for the resonance region, in the first approximation, the frequency equation of forced oscillations of the rotor can be written as (8) [13]:

(14) $$L\left(\varphi^{\prime }\right)-q\varphi^{\prime }-\left(\mu_{1}+0.75\mu_{3}\omega_{n}^{2}a^{2}\right)\omega_{n}a^{2}=0,$$

Where a is the amplitude of the stationary oscillations of the rotor. The roots of Eq. (14) are the intersection points of the graph $L(\varphi^{\prime })$ - motor performance characteristic and graph $S(\varphi^{\prime })$ - moments of forces of resistance to the motor rotor rotational motion and forces of the rotor oscillatory motion damping:

(15) $$S\left(\varphi^{\prime }\right)=q\varphi^{\prime }+\left(\mu_{1}+0.75\mu_{3}\omega_{n}^{2}a^{2}\right)\omega_{n}a^{2}.$$

Here $a^{2}$ can be find as the root of cubic equation – equations of the frequency response of stationary rotor oscillations (9): $\left\{{\left(\mu_{1}\omega_{n}+0.75\mu_{3}\omega_{n}^{3}a^{2}\right)}^{2}+{\left[0.75C_{3}a^{2}+2\omega_{n}\left(\omega_{n}-\varphi^{\prime }\right)\right]}^{2}\right\}a^{2}={\left(e{\varphi^{\prime }}^{2}\right)}^{2}$:

(16) $$\begin{array}{ccc} a^{2} & = & \sqrt[{3}]{-\frac{1}{2}\left[2{\left(\frac{a_{0}}{3}\right)}^{3}-\frac{a_{0}b_{0}}{3}+c_{0}\right]+\sqrt{{\left[\frac{1}{3}\left(-\frac{a_{0}^{2}}{3}+b_{0}\right)\right]}^{3}+{\left\{\frac{1}{2}\left[2{\left(\frac{a_{0}}{3}\right)}^{3}-\frac{a_{0}b_{0}}{3}+c_{0}\right]\right\}}^{2}}}\\ & & +\sqrt[{3}]{-\frac{1}{2}\left[2{\left(\frac{a_{0}}{3}\right)}^{3}-\frac{a_{0}b_{0}}{3}+c_{0}\right]-\sqrt{{\left[\frac{1}{3}\left(-\frac{a_{0}^{2}}{3}+b_{0}\right)\right]}^{3}+{\left\{\frac{1}{2}\left[2{\left(\frac{a_{0}}{3}\right)}^{3}-\frac{a_{0}b_{0}}{3}+c_{0}\right]\right\}}^{2}}}-\frac{a_{0}}{3}, \end{array}$$

where

(17) $$a_{0}=\frac{1.5\mu_{1}\mu_{3}\omega_{n}^{4}+3C_{3}\omega_{n}\left(\omega_{n}-\varphi^{\prime }\right)}{0.5625\left(\mu_{3}^{2}\omega_{n}^{6}+C_{3}^{2}\right)},\;b_{0}=\frac{\mu_{1}^{2}\omega_{n}^{4}+4\omega_{n}^{2}{\left(\omega_{n}-\varphi^{\prime }\right)}^{2}}{0.5625\left(\mu_{3}^{2}\omega_{n}^{6}+C_{3}^{2}\right)},\;c_{0}=-\frac{{\left(e{\varphi^{\prime }}^{2}\right)}^{2}}{0.5625\left(\mu_{3}^{2}\omega_{n}^{6}+C_{3}^{2}\right)}.$$

In the direct integration of Eq. (3), taking into account the expression (15)-(17), the following rotor parameters were used: the eccentricity of the disk mass e=0.0346 and its polar moment of inertia $I_{P1}$=0.021. When converting the parameters to the dimensionless form, measured values of linear and dynamic parameters were used, borrowed from the experimental installation used in the work [12,13]. The values of the coefficient of nonlinear rigidity of the support $C_{3}$=0.1 and the resonant frequency $\omega_{n}$≈1 were adopted for the clarity of the obtained results.

When selecting the baseline conditions we use the analytically found expressions for α,β,φ and $\varphi^{\prime }$ with consideration to the odd harmonics in accordance with formulas (11)–(13) and the values of amplitude and initial phase of the main harmonic of the stationary oscillations found based on the amplitude and phase-frequency characteristics (9) and (10).

Thus, we take the initial conditions as at $\bar{t}$=0: $\alpha_{0}$=0.8918, $\beta_{0}$=−0.6092, $\varphi_{0}$=0.2599, ${\alpha^{\prime }}_{0}$=−0.6275, ${\beta^{\prime }}_{0}$=0.9175, ${\varphi^{\prime }}_{0}$=0.2599 at *μ*_3_=0.01 and *μ*_1_=0.01; $\alpha_{0}$=0.6847, $\beta_{0}$=−0.7960, $\varphi_{0}$=0.2607, ${\alpha^{\prime }}_{0}$=0.8199, ${\beta^{\prime }}_{0}$=0.7053, ${\varphi^{\prime }}_{0}$=0.0812 at *μ*_3_=0.02 and *μ*_1_=0.01; $\alpha_{0}$=0.0097, $\beta_{0}$=−0.9700, $\varphi_{0}$=0.0045, ${\alpha^{\prime }}_{0}$=1.0039, ${\beta^{\prime }}_{0}$=0.010, ${\varphi^{\prime }}_{0}$=0.4624 at *μ*_3_=0.04 and *μ*_1_=0.01. These values of the initial conditions correspond to points up to the bistable region of the resonance curves at different values of the nonlinear cubic damping coefficient.

The integration of the system of Eq. (3) with expressions (15)-(17) and baseline conditions was done using MatLab and the developed blocks and subblocks of MatLab-Simulink (R2021a (9.10.0.1602886) 64-bit (win 61) February 17, 2021).

## Results

**Fig. 2:**
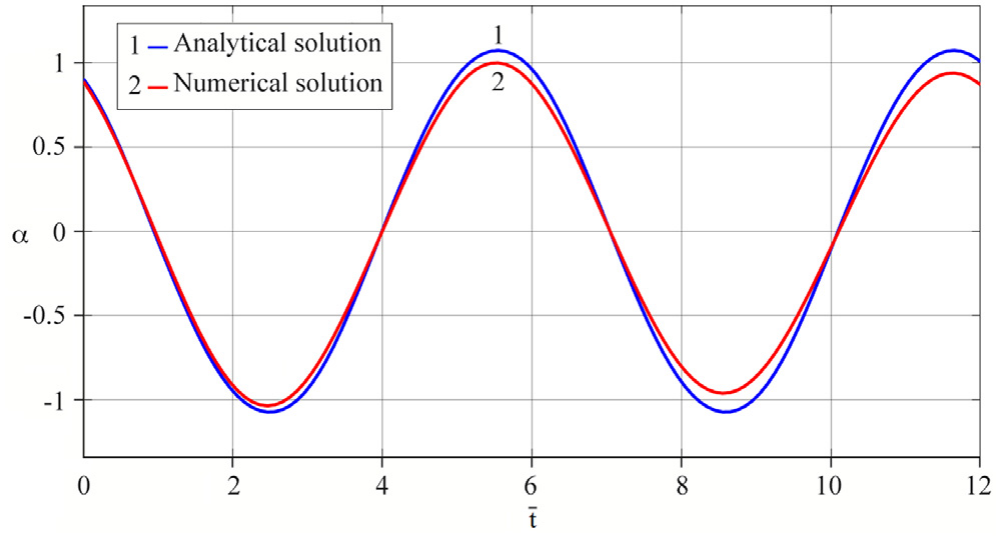
Graphical dependencies $\alpha =\alpha (\bar{t})$ plotted based on analytical and direct solutions of equations of motion (3) for μ_3_=0.01 and μ_1_=0.01.

**Fig. 3:**
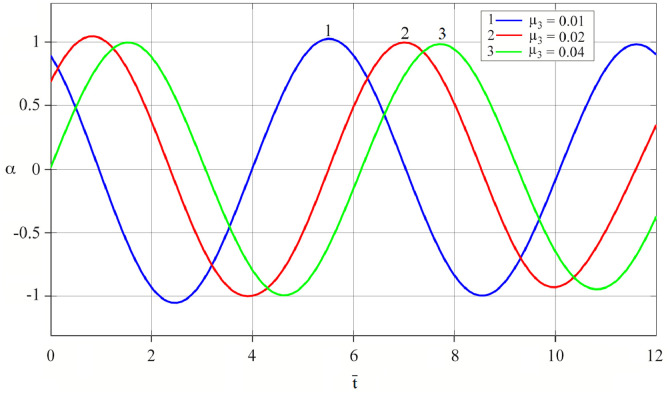
Graphical dependencies $\alpha =\alpha (\bar{t})$ plotted based on analytical and direct solutions of equations of motion (3) for various values of μ_3_ and μ_1_=0.01.

**Fig. 4:**
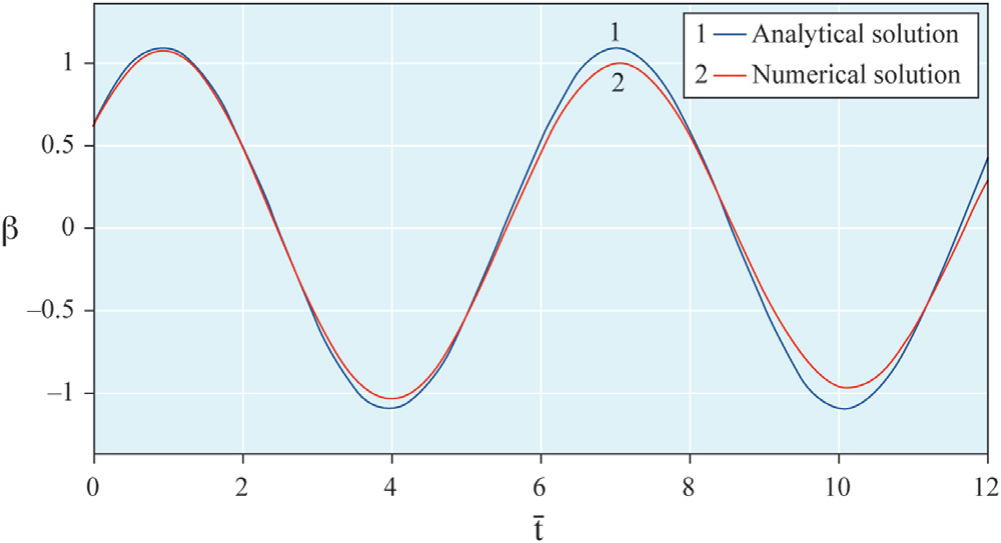
Graphical dependencies $\beta =\beta (\bar{t})$ plotted based on analytical and direct solutions of equations of motion (3) for μ_3_=0.01 and μ_1_=0.01.

**Fig. 5:**
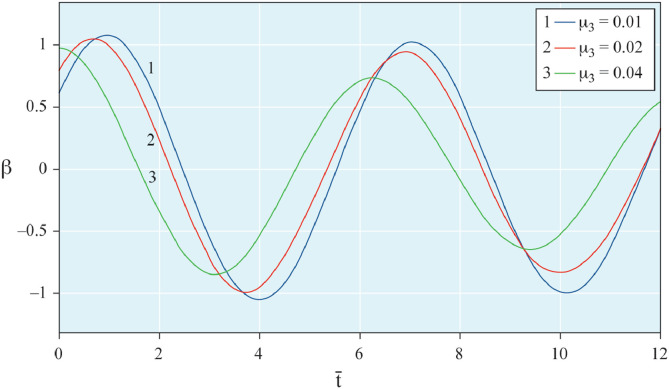
Graphical dependencies $\beta =\beta (\bar{t})$ plotted based on analytical and direct solutions of equations of motion (3) for various values of μ_3_ and μ_1_=0.01.

**Fig. 6:**
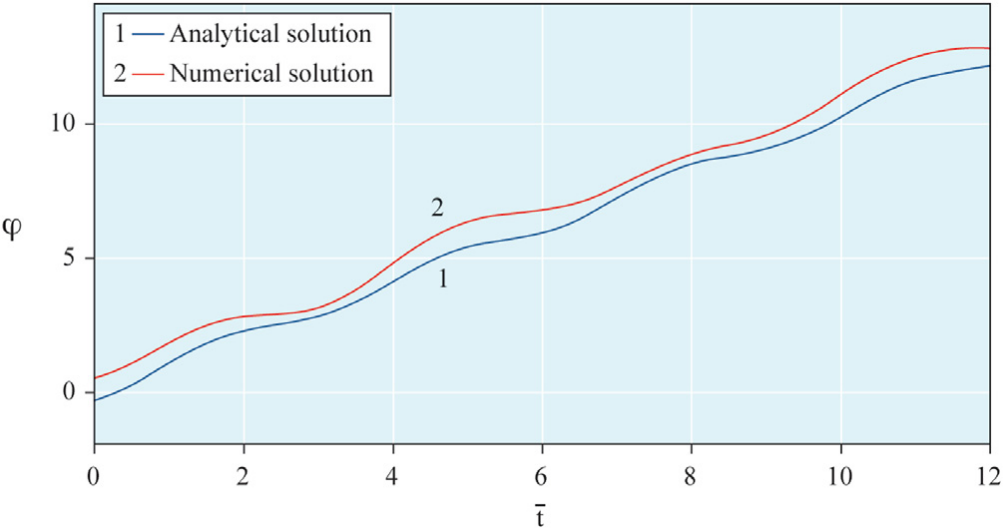
Graphical variations $\varphi =\varphi (\bar{t})$ plotted based on analytical and direct solutions of equations of motion (3) for μ_3_=0.01 and μ_1_=0.01.

**Fig. 7:**
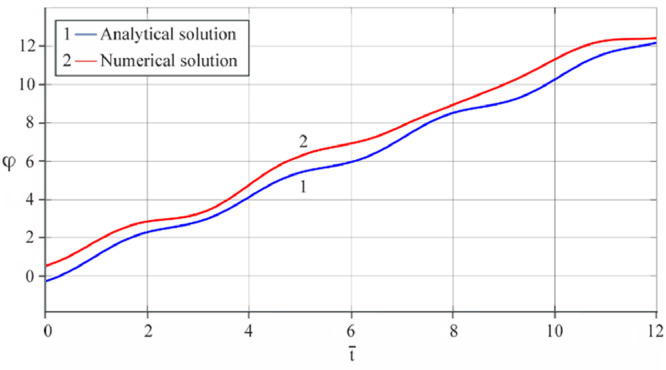
Graphical variations $\varphi^{\prime }=\varphi^{\prime }\left(\bar{t}\right)$ plotted based on analytical and direct solutions of equations of motion (3) for various values of μ_3_=0.04 and μ_1_=0.01.

**Fig. 8:**
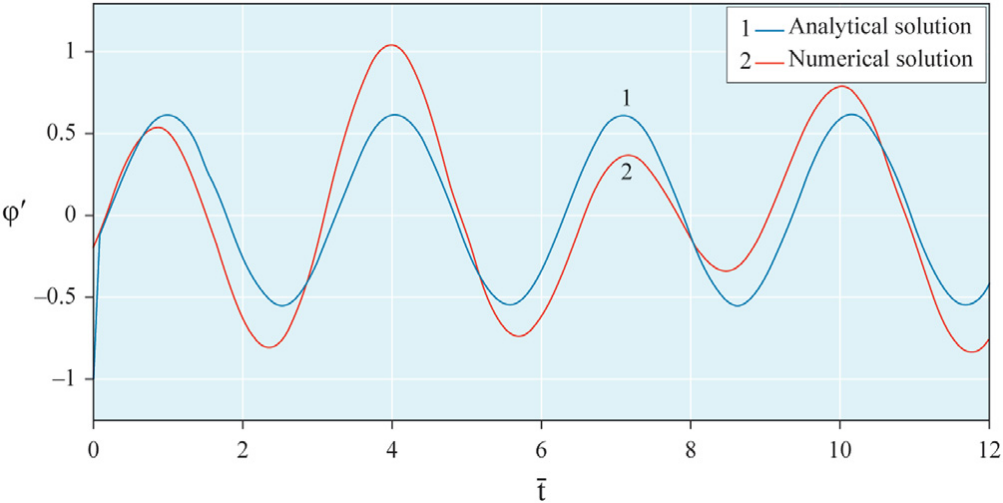
Graphical variations $\varphi^{\prime }=\varphi^{\prime }\left(\bar{t}\right)$ plotted based on analytical and direct solutions of equations of motion (3) for various values of μ_3_=0.01 and μ_1_=0.01.

**Fig. 9:**
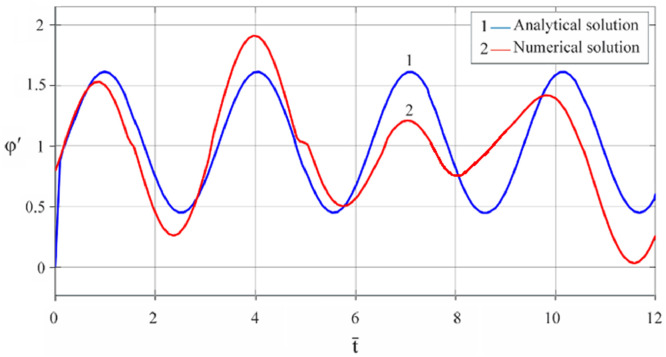
Graphical variations $\varphi^{\prime }=\varphi^{\prime }\left(\bar{t}\right)$ plotted based on analytical and direct solutions of equations of motion (3) for various values of μ_3_=0.04 and μ_1_=0.01.

The results of analytical simulation based on Eqs. (11)–(13) taking into account the expressions of frequency characteristics (9) and (10), and numerical simulation based on Eqs. (3), (15)–(17) in comparative form are shown on the Fig. 2, Fig. 3, Fig. 4, Fig. 5, Fig. 6, Fig. 7. Comparison of the oscillograms $\alpha =\alpha \left(\bar{t}\right),\beta =\beta \left(\bar{t}\right)$ at *μ*_3_=0.01, and *μ*_1_=0.01 and $\varphi =\varphi \left(\bar{t}\right),\varphi^{\prime }=\varphi^{\prime }\left(\bar{t}\right)$ at  *μ*_3_=0.01, 0.04 and *μ*_1_=0.01, plotted based on the numerical results with similar oscillograms plotted based on the analytical formulas $\alpha ,\beta ,\varphi ,\varphi^{\prime }$ and frequency responses (Figs. 2, 4, 6–9) indicates that the results of analytical and numerical solutions of equations of motion are close to each other, which proves the acceptability of the presented method of numerical solution. From dependency graphs $\alpha =\alpha (\bar{t})$ и $\beta =\beta (\bar{t})$ shown on the Figs. 3 and 5 respectively, plotted based on numerical solutions of Eq. (3) for various values of *μ*_3_=0.01, 0.02, 0.04 and for constant values *μ*_1_=0.01, the joint damping effect of values *μ*_3_ and *μ*_1_ is clearly evident in the region of resonance, which supports the results of analytical solutions of the equations of motion (3) [13].

**Fig. 10:**
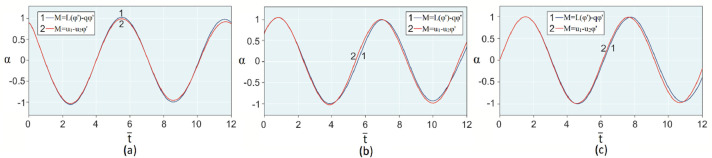
Oscillograms $\alpha =\alpha (\bar{t})$ plotted based on direct solutions of equations of motion (3) for μ_1_=0.01 and for various values of μ_3_ and $u_{1}$: (a) 0.01 and 1.304, (b) 0.02 and 1.314, (c) 0.04 and 1.323.

**Fig. 11:**
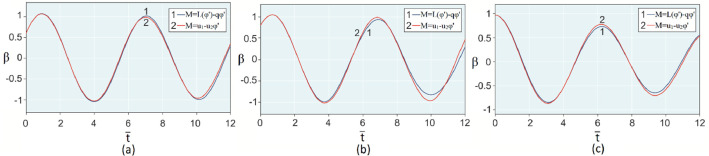
Oscillograms $\beta =\beta (\bar{t})$ plotted based on direct solutions of equations of motion (3) for μ_1_=0.01 and for various values of μ_3_ and $u_{1}$: (a) 0.01 and 1.304, (b) 0.02 and 1.314, (c) 0.04 and 1.323.

**Fig. 12:**
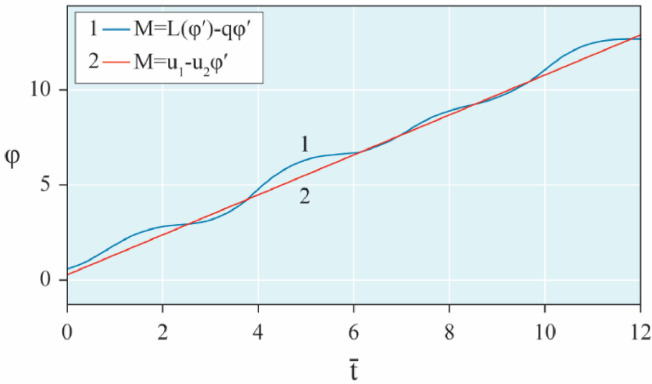
Graphical variations $\varphi =\varphi (\bar{t})$ plotted based on numerical modeling of equations of motion (3) for μ_1_=0.01, μ_3_=0.01 and $u_{1}$=1.304.

**Fig. 13:**
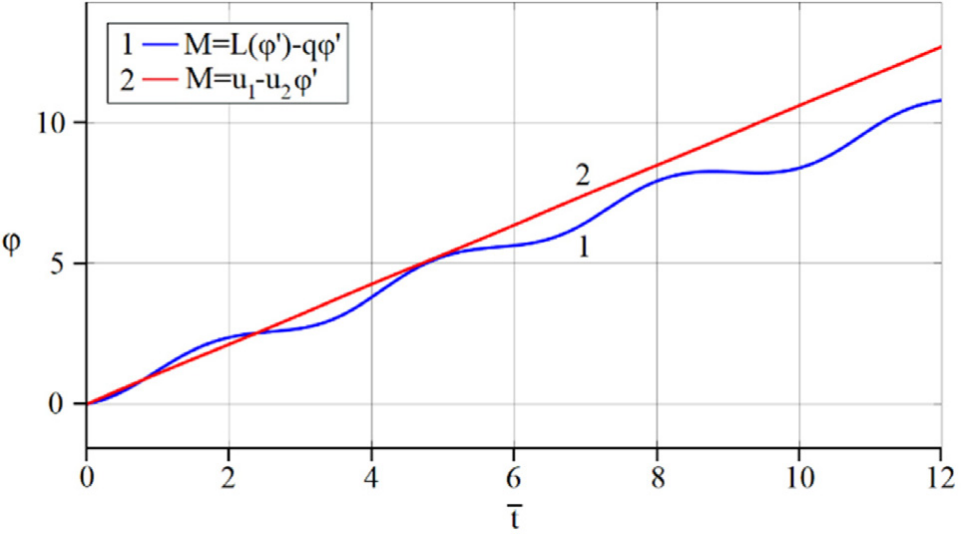
Graphical variations $\varphi =\varphi (\bar{t})$ plotted based on numerical modeling of equations of motion (3) for μ_1_=0.01, μ_3_=0.04 and $u_{1}$=1.323.

**Fig. 14:**
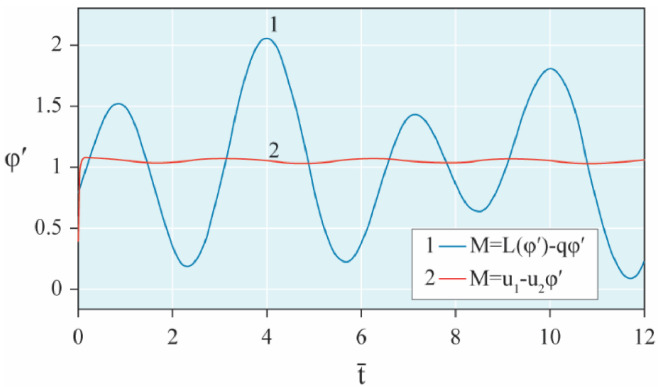
Graphical variations $\varphi^{\prime }=\varphi^{\prime }\left(\bar{t}\right)$ plotted based on numerical modeling of equations of motion (3) for μ_1_=0.01, μ_3_=0.01 and $u_{1}$=1.304.

**Fig. 15:**
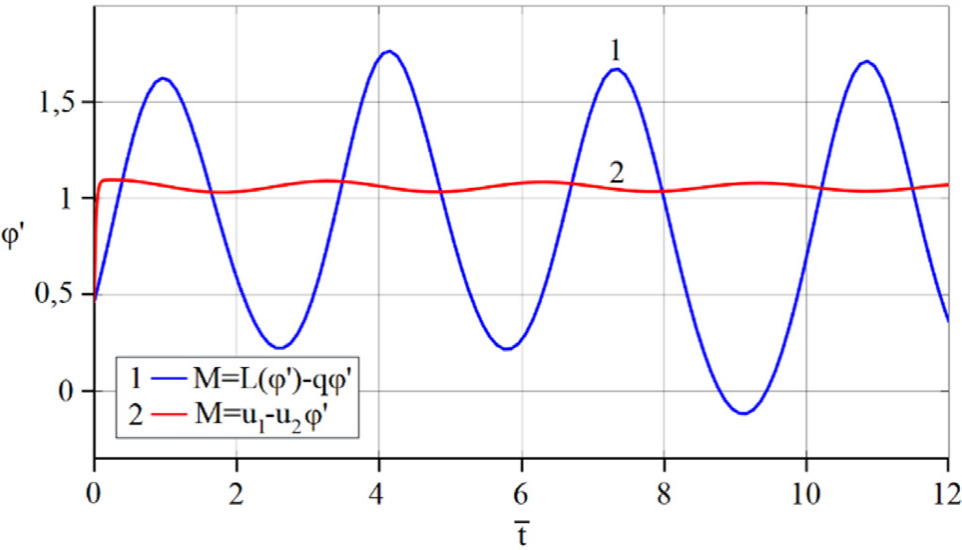
Graphical variations $\varphi^{\prime }=\varphi^{\prime }\left(\bar{t}\right)$ plotted based on numerical modeling of equations of motion (3) for μ_1_=0.01, μ_3_=0.04 and $u_{1}$=1.323.

Now let us solve the system of differential equations of motion (3) numerically using MatLab-Simulink for the known performance characteristic of the electric motor: $M\left(\varphi^{\prime }\right)=u_{1}-u_{2}\varphi^{\prime },u_{2}=1.245$ [48]. Then, let us compare the results with the results of direct simulation of the system of Eq. (3) for the general form of motor performance characteristic representation: $M=L\left(\varphi^{\prime }\right)-q\varphi^{\prime }$ (Fig. 10, Fig. 11, Fig. 12, Fig. 13, Fig. 14, Fig. 15). From Fig. 10, Fig. 11, Fig. 12, Fig. 13, Fig. 14, Fig. 15 the closeness of the results of direct modeling of the system of Eq. (3) for $M\left(\varphi^{\prime }\right)=u_{1}-u_{2}\varphi^{\prime }$ and for $M=L\left(\varphi^{\prime }\right)-q\varphi^{\prime }$ is clear evident. The difference of these results in the oscillograms $\varphi^{\prime }=\varphi^{\prime }\left(\bar{t}\right)$ (Figs 14 and 15) is explained with a predetermined straight-line performance characteristic of the electric motor. The approximation and limitedness of the proposed method is also confirmed by an increase in the divergence of the results of analytical and numerical solutions to the equations of motion, the results of direct modeling of the equations of motion with a common and given form of the engine characteristic relative to $\varphi^{\prime }$ (Fig. 9) and φ (Fig. 13), respectively, with a large value of the nonlinear damping coefficient, *μ*_3_, equal to 0.04 over time.

**Fig. 16:**
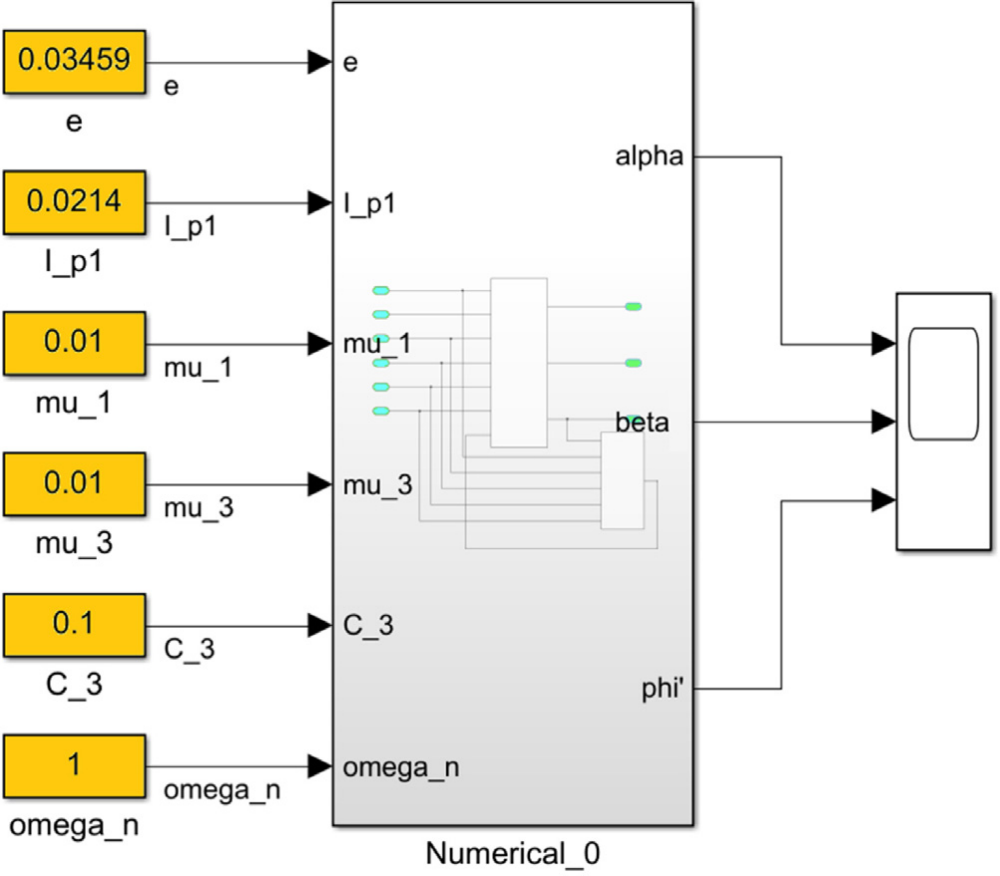
Direct modeling of differential equations of motion (3).

**Fig. 17:**
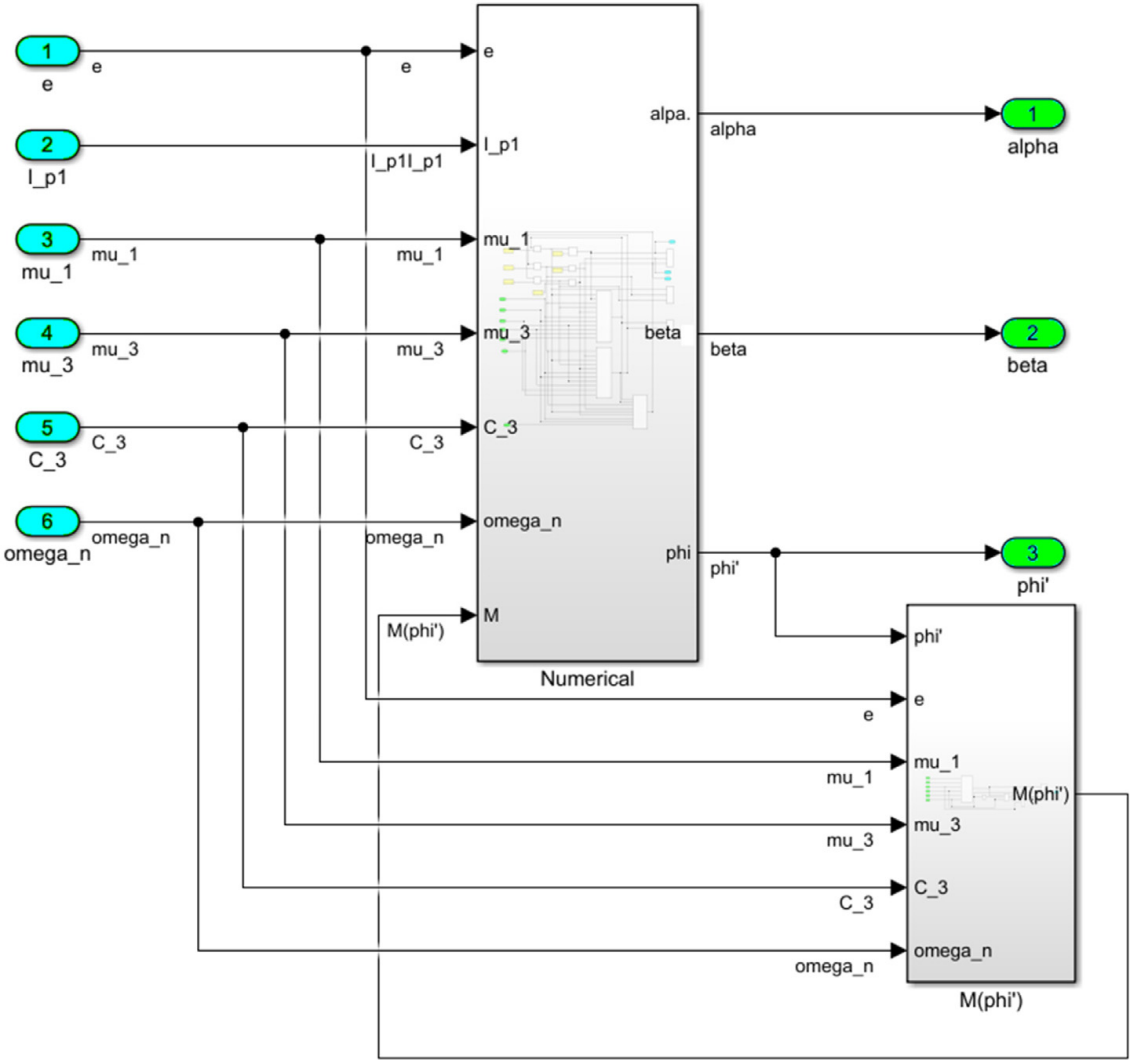
The connection of the Numerical block with the dynamic torque block of the motor M(phi').

**Fig. 18:**
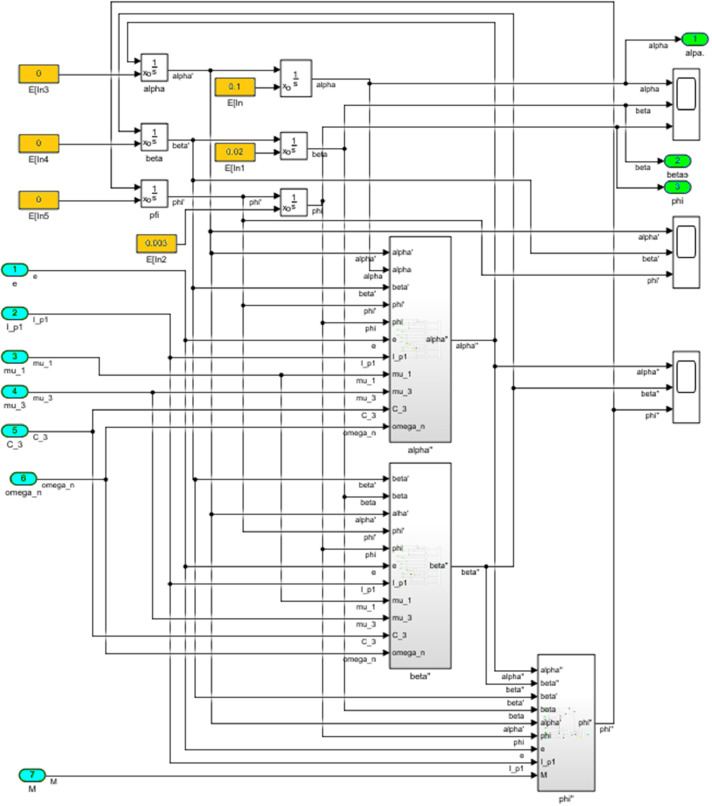
The structural content of the Numerical block.

**Fig. 19:**
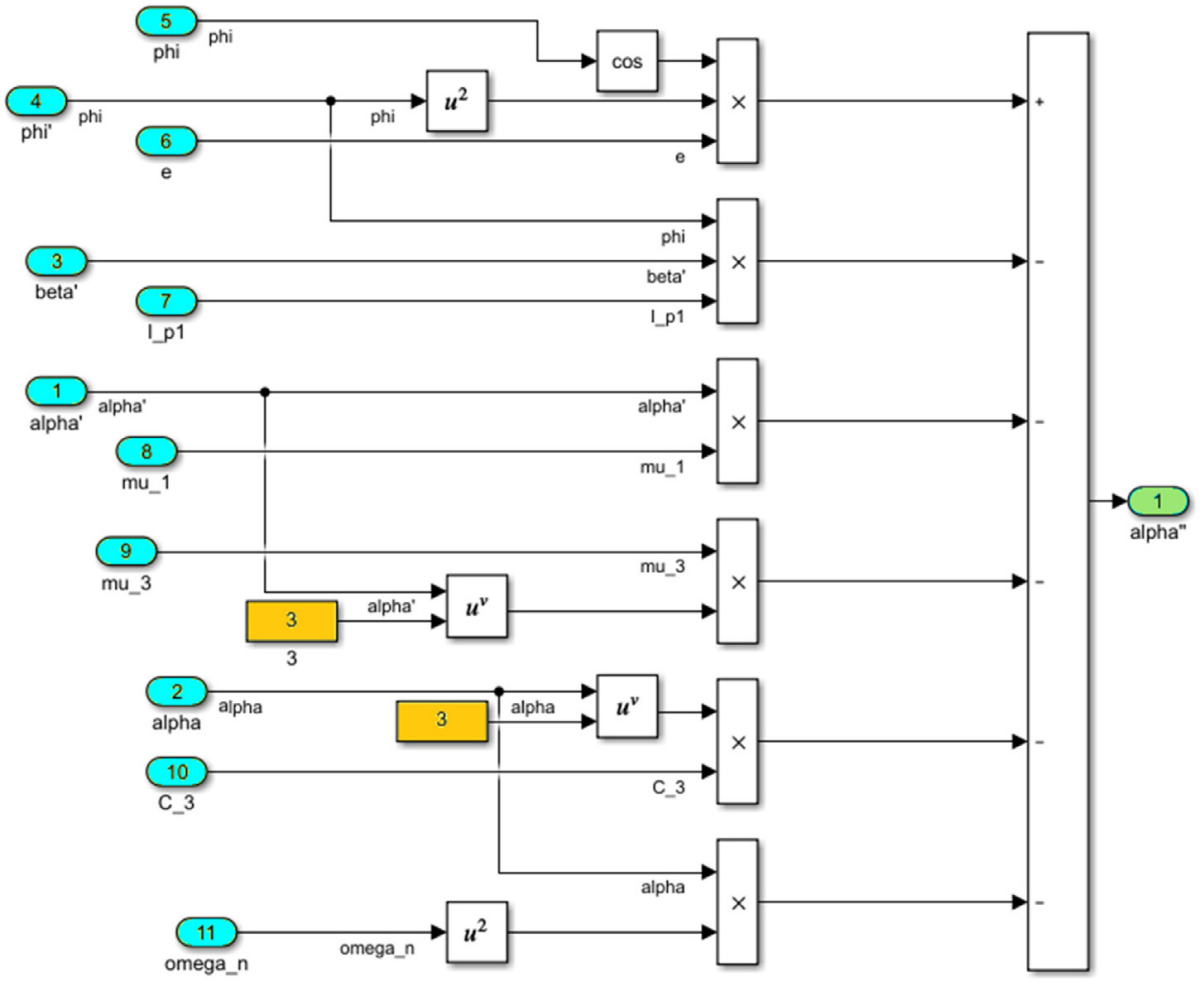
A subblock with the contents for the computational operation of the right side of the first equation of the system (3).

**Fig. 20:**
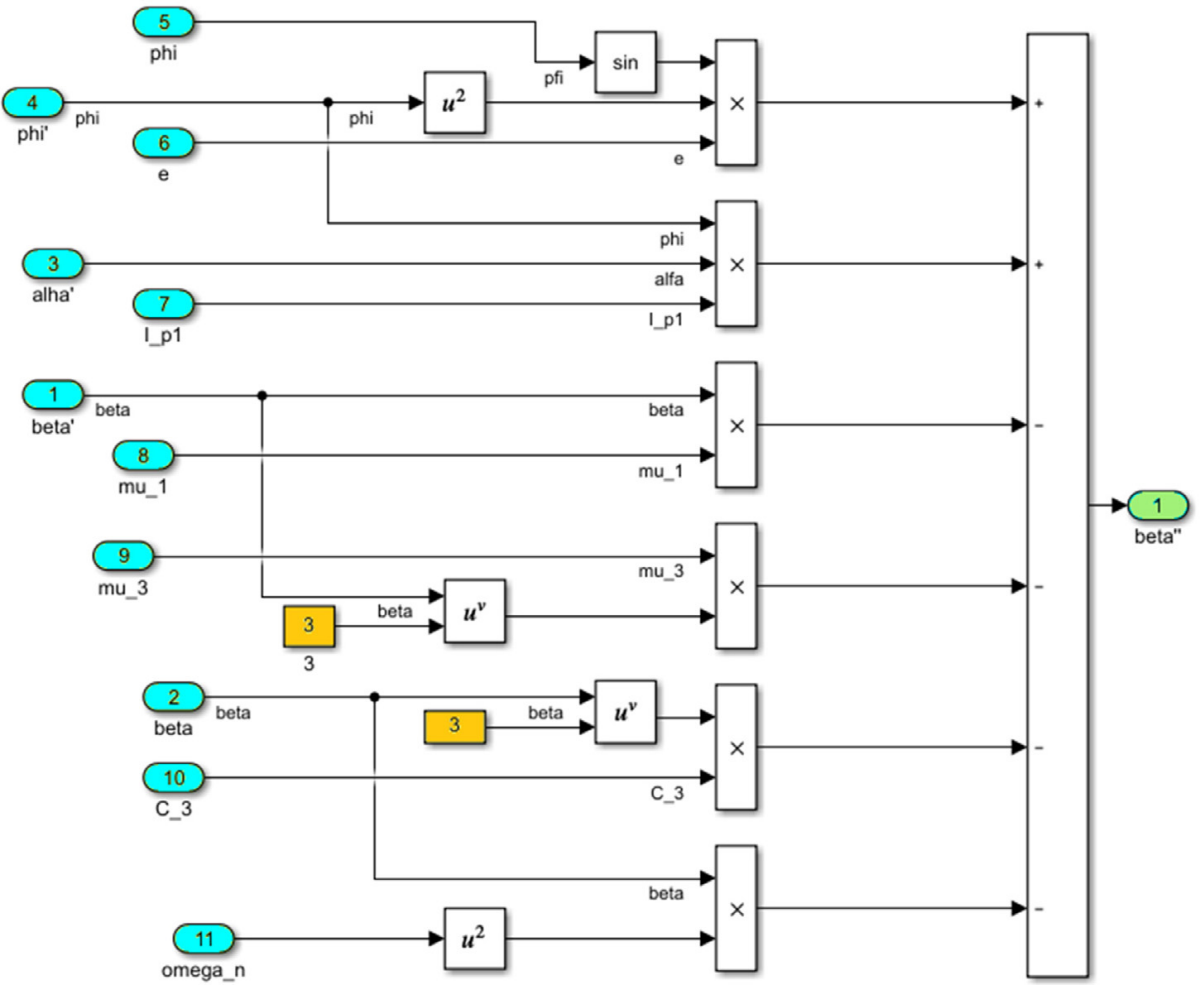
A subblock with the contents for the computational operation of the right side of the second equation of the system (3).

**Fig. 21:**
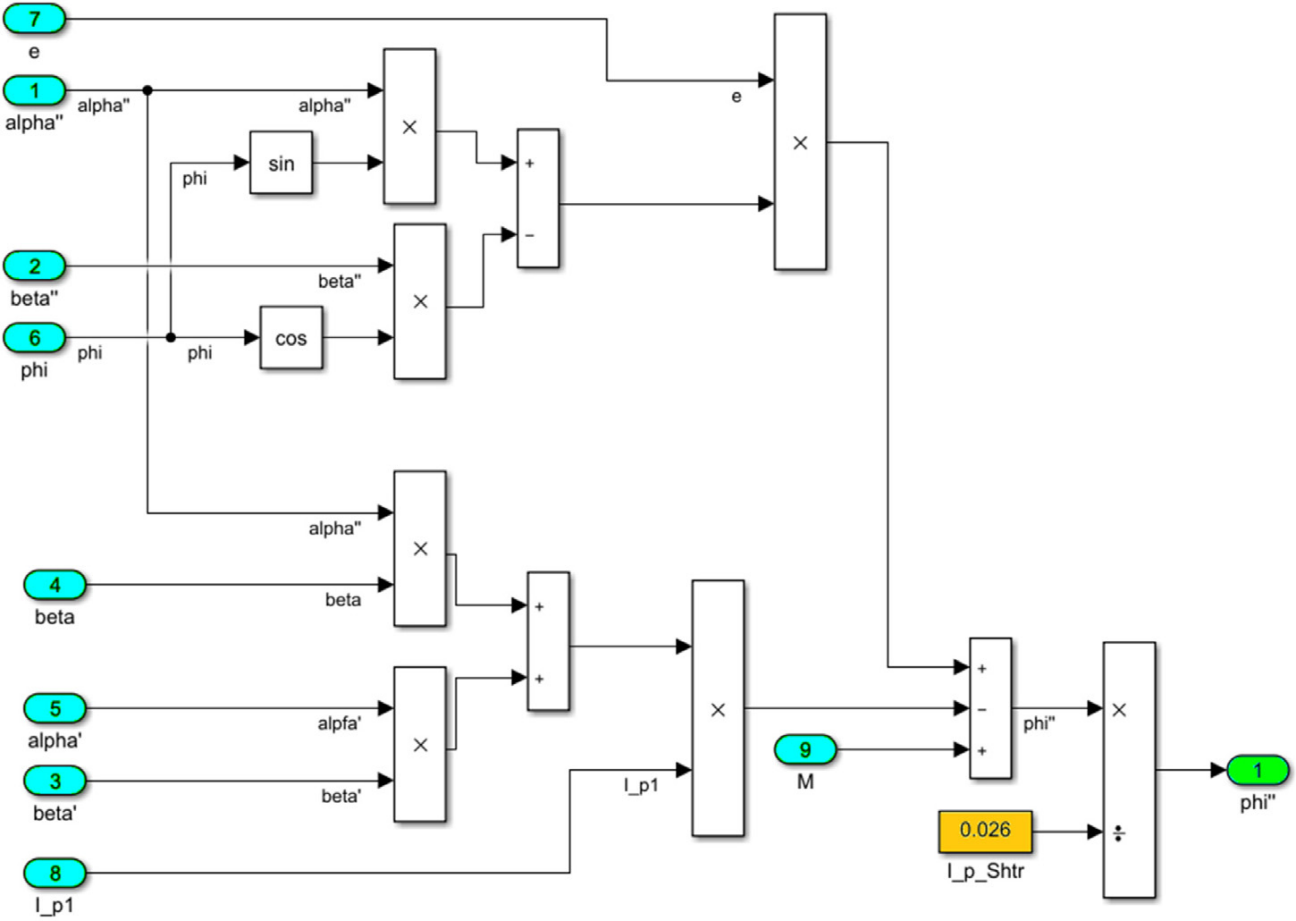
A subblock with the contents for the computational operation of the right side of the third equation of the system (3).

The numerical solution of the differential equations of motion of the rotor (3) taking into account (15)-(17) in the form of a Simulink model is shown in Fig. 16. The model includes a Numerical block and a Dynamic moment block M(phi') (see Fig. 17). The structure of the Numerical block is shown in Fig. 18, in its content, the integration of a system of differential Eq. (3) is performed. In the structure of the Numerical block there are alpha", beta" and phi" subblocks for the computational operation of the right-hand sides of differential Eq. (3). Their contents are shown in Fig. 19, Fig. 20, Fig. 21.

**Fig. 22:**
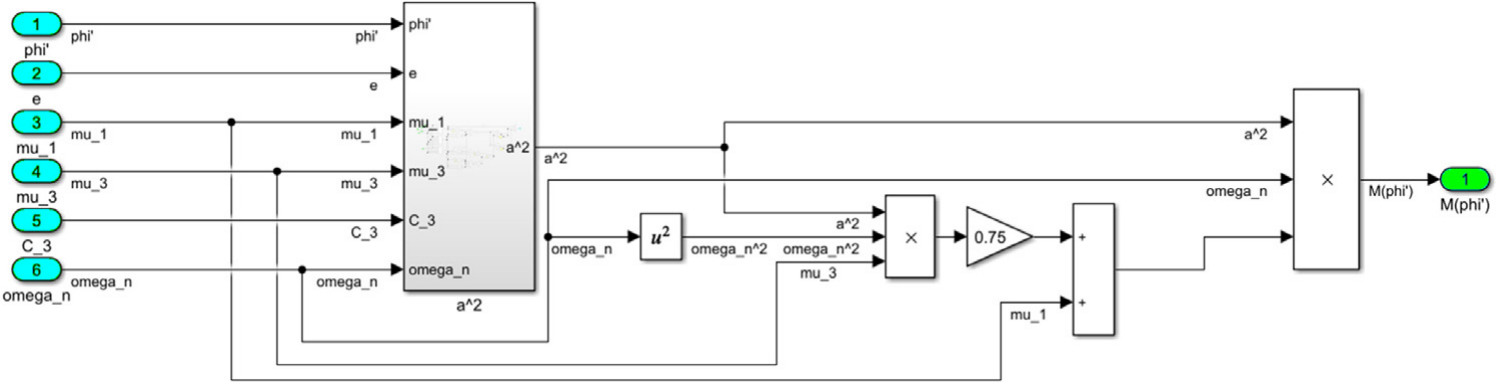
The structural content of M(phi') block.

**Fig. 23:**
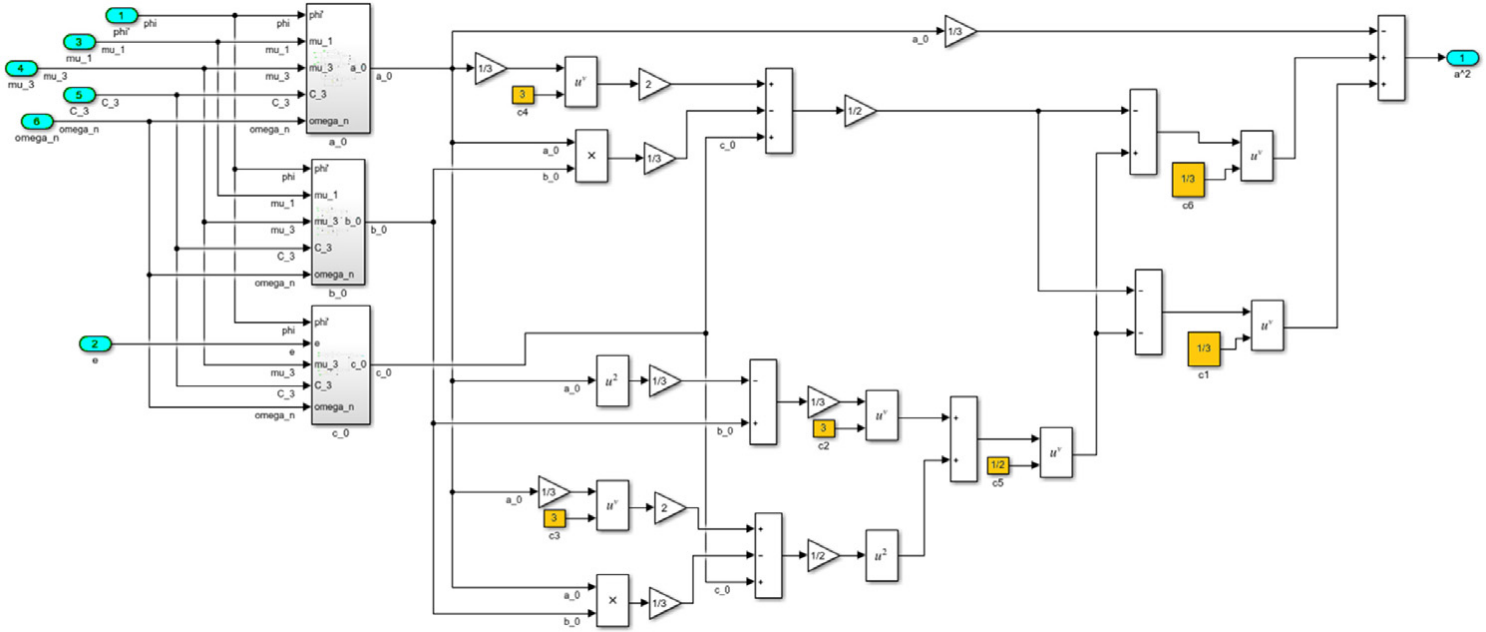
The structural content of a^2 subblock.

**Fig. 24:**
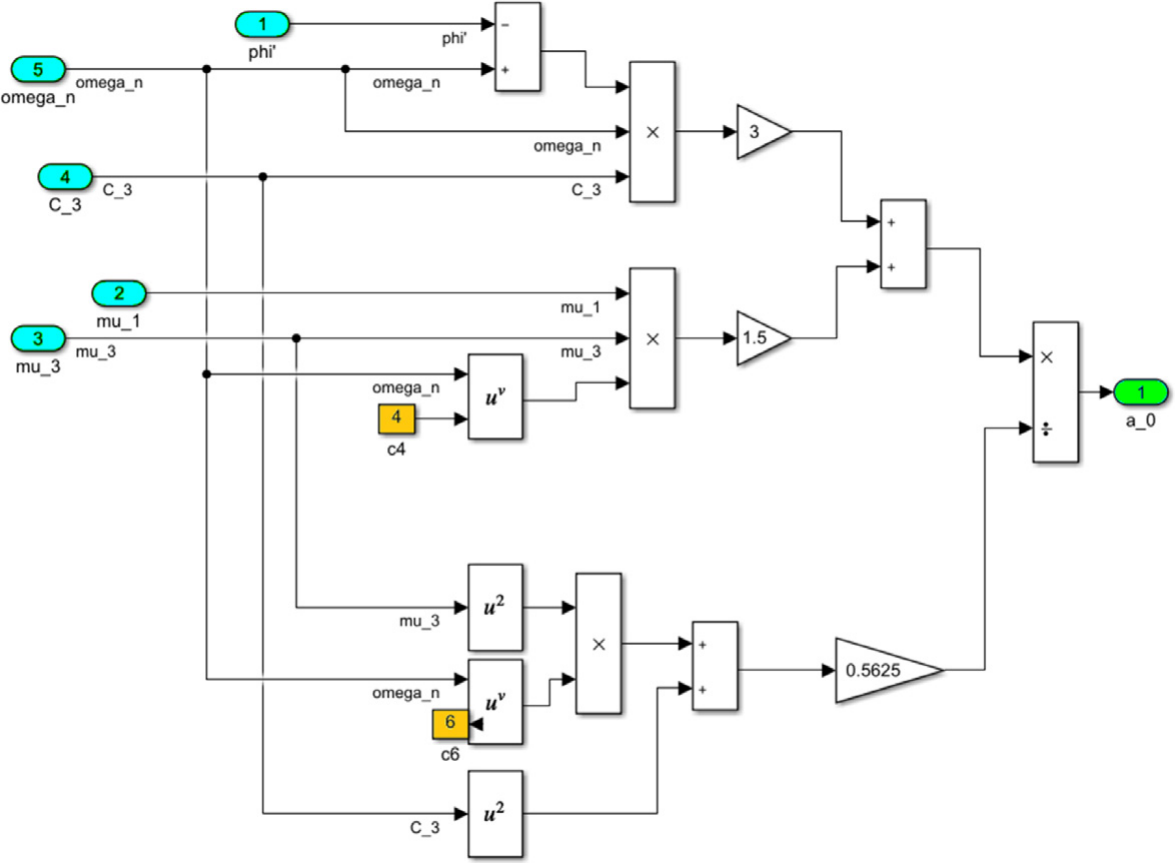
The structural content of a0 subblock.

**Fig. 25:**
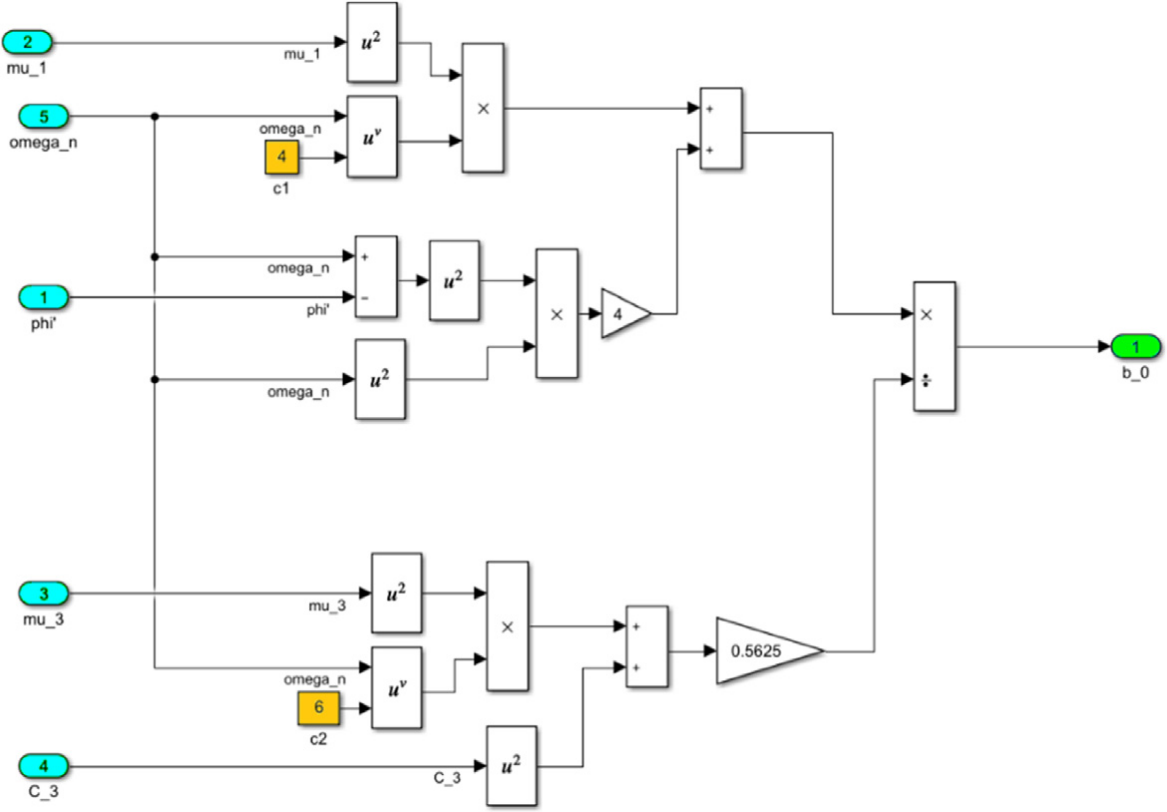
The structural content of b0 subblock.

**Fig. 26:**
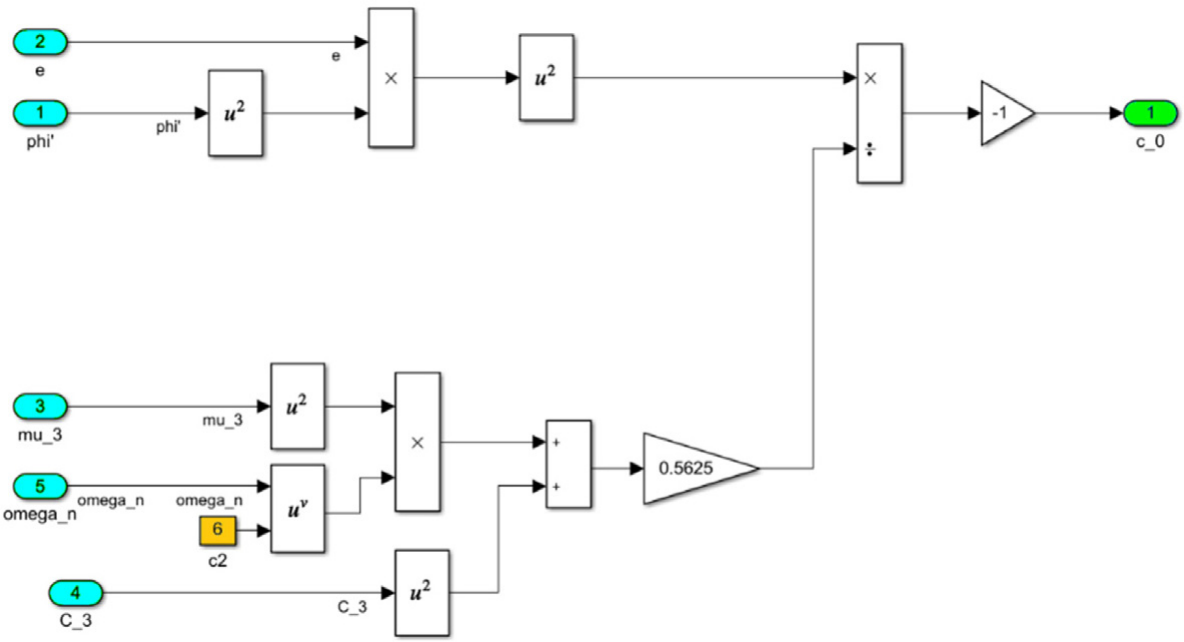
The structural content of c0 subblock.

The block M(phi') with its contents is shown in Fig. 22, where the dynamic torque of the motor M(φ’) = L(φ’)-q φ’ is calculated. This block also contains a subblock a^2 for the computational operation of the values of a^2 according to the formula (16). The content of subblock a^2 is shown on the Fig. 23. In turn, the subblock a^2 has subblocks a_0, b_0 and c_0, which are designed to perform the computational operation of the coefficients *a_0_, b_0_, с_0_*. Their contents are shown in Figs. 24, 25 and 26, respectively.

The software for reproducing the method is given in [49] and executed by MathLab-Simulink package (R2021a (9.10.0.1602886) 64-bit (win 61) 17 February 2021).

## Discussion

Therefore, when the motor performance characteristic is unknowns, a method of numerical solution of nonlinear differential equations of motion of a nonideal gyroscopic rigid rotor system with linear and nonlinear cubic damping and nonlinear rigidity of the elastic support is developed. The essence of the method is described below. As part of the analytical solution using the Bogolyubov–Krylov small perturbation theory method [13,15,33], we have obtained the expressions for the amplitude and phase-frequency characteristics of the main harmonic of the stationary oscillations, which are structurally unrelated to the motor performance characteristics, and we have also separately obtained the frequency equation, which connects the dynamic moment of the engine with the oscillation parameters, restoring and damping characteristics of the rotor system, which proves the relation of the rotor system to the energy source. The proof is that the analytical dynamic analysis of the paper [13] revealed a change in the speed of rotation of the motor shaft (frequency of oscillation of the rotor) according to a harmonic law around a certain average value Ω (stationary value of the shaft speed), the effect of nonlinear stiffness coefficients, linear damping, joint linear and nonlinear cubic damping on the variation of the motor shaft speed (rotor oscillation frequency), on its amplitude and the rotor precession frequency, and on the non-uniformity of the motor shaft speed (rotor). Despite the interaction of the rotor system with the energy source, the nonlinear differential equations of motion of the rotor-motor system, when the performance characteristics of the energy source is unknown, become numerically unsolvable. In this case, the expression found from the frequency equation of forced stationary oscillations assuming that $\varphi^{\prime \prime }\ll {\varphi^{\prime }}^{2}$ with the replacement of the stationary rotation angular velocity with the derivative of the shaft angle of rotation $\varphi^{\prime }$ is used as the motor performance characteristic. The plausibility of the proposed method was checked by comparing first the results of direct simulation using the MatLab-Simulink package and the results of analytical solution of the nonlinear differential equations of rotor motion by the Bogolyubov–Krylov small perturbation theory method [13,15,33], and then the results of numerical solutions with the unknown characteristic of the energy source presented in general form $M=L\left(\varphi^{\prime }\right)-q\varphi^{\prime }$ and the straight-line DC motor performance characteristic $M\left(\varphi^{\prime }\right)=u_{1}-u_{2}\varphi^{\prime }$. Graphical results in the form of oscillograms of angular coordinates $\alpha =\alpha \left(\bar{t}\right),\beta =\beta \left(\bar{t}\right)$ and $\varphi =\varphi (\bar{t})$ demonstrate the validity of the stated assumption of the proposed method. The method also proves the suppression of the amplitude near resonance oscillations of angular coordinatesα and β by the influence of joint linear and nonlinear cubic damping. The divergence of the oscillograms $\varphi^{\prime }=\varphi^{\prime }\left(\bar{t}\right)$ potted for $M=L\left(\varphi^{\prime }\right)-q\varphi^{\prime }$ and for $M\left(\varphi^{\prime }\right)=u_{1}-u_{2}\varphi^{\prime }$ is explained by the setting of a straight-line DC motor characteristic, which at a given value of the control parameter $u_{1}$ will affect the regularity $\varphi^{\prime }=\varphi^{\prime }\left(\bar{t}\right)$. A noticeable difference in the compared results relative to the values $\varphi^{\prime }$ (see Fig. 9) and φ (see Fig. 13) is also observed with a large value of the coefficient of nonlinear cubic damping *μ*_3_ =0.04 over time. Consequently, the proposed method is valid with respect to direct numerical solutions of nonlinear differential equations of motion to determine the angular coordinates $\alpha =\alpha \left(\bar{t}\right),\beta =\beta \left(\bar{t}\right)$ and $\varphi =\varphi (\bar{t})$, but limited in that it is used for the first approximation and weak nonlinear oscillations in the resonance region, where the shaft rotation speed is of the order of the oscillating system natural frequency.

## Conclusion

This study describes a developed method for direct modeling of nonlinear differential equations of motion of a nonideal gyroscopic rigid rotor system with linear and nonlinear cubic damping and nonlinear stiffness of the elastic support, when the energy source characteristic is unknown.

In this method, the motor drive characteristic is replaced by its expression found from the frequency equation of forced stationary oscillations, assuming that the angular acceleration is many times less than the square of the angular speed of rotation, replacing the angular speed of stationary rotation with the derivative of the angle of rotation of the shaft.

The results of numerical solution of the nonlinear differential equations of motion are well consistent with the results of the analytical solution, while the results of direct simulation of the rotor system dynamics for unknown characteristic of the energy source and for the straight-line drive characteristic of DC motor drive are consistently close to each other (relative to the angular coordinates of the rotor shaft).

Numerical dynamic analysis demonstrates the suppression of rotor near-resonance oscillation amplitude by linear and nonlinear cubic damping of the elastic support.

The method is used for direct solution of nonlinear differential equations of motion with respect to angular coordinates of the shaft, but is limited in that it is intended for the first approximation and weak nonlinear vibrations in the resonance region, where the shaft rotation speed is of the order of the natural frequency of the oscillating system.

Co-Submission: Co-submissiong, 10.1016/j.ymssp.2021.108773.

## Funding

This research has been/was/is funded by the Science Committee of the 10.13039/501100012190Ministry of Science and Higher Education of the Republic of Kazakhstan (Grants No. AP15473701, AP08856763).

## Declaration of Competing Interest

The authors declare that they have no known competing financial interests or personal relationships that could have appeared to influence the work reported in this paper.

## Data Availability

No data was used for the research described in the article.
